# Stepped wedge cluster randomised trials: a review of the statistical methodology used and available

**DOI:** 10.1186/s12874-016-0176-5

**Published:** 2016-06-06

**Authors:** D. Barker, P. McElduff, C. D’Este, M. J. Campbell

**Affiliations:** School of Medicine and Public Health, Faculty of Health, CCEB, HMRI Building, Level 4 West, University of Newcastle, University Drive, Callaghan, NSW 2308 Australia; National Centre for Epidemiology and Population Health, Research School of Population Health, Australian National University, Canberra, ACT 0200 Australia; Medical Statistics Group, ScHARR, University of Sheffield, Sheffield, UK

**Keywords:** Stepped wedge, Cluster randomised, Statistical methodology

## Abstract

**Background:**

Previous reviews have focussed on the rationale for employing the stepped wedge design (SWD), the areas of research to which the design has been applied and the general characteristics of the design. However these did not focus on the statistical methods nor addressed the appropriateness of sample size methods used.This was a review of the literature of the statistical methodology used in stepped wedge cluster randomised trials.

**Methods:**

Literature Review. The Medline, Embase, PsycINFO, CINAHL and Cochrane databases were searched for methodological guides and RCTs which employed the stepped wedge design.

**Results:**

This review identified 102 trials which employed the stepped wedge design compared to 37 from the most recent review by Beard et al. 2015. Forty six trials were cohort designs and 45 % (*n* = 46) had fewer than 10 clusters. Of the 42 articles discussing the design methodology 10 covered analysis and seven covered sample size. For cohort stepped wedge designs there was only one paper considering analysis and one considering sample size methods. Most trials employed either a GEE or mixed model approach to analysis (*n* = 77) but only 22 trials (22 %) estimated sample size in a way which accounted for the stepped wedge design that was subsequently used.

**Conclusions:**

Many studies which employ the stepped wedge design have few clusters but use methods of analysis which may require more clusters for unbiased and efficient intervention effect estimates. There is the need for research on the minimum number of clusters required for both types of stepped wedge design. Researchers should distinguish in the sample size calculation between cohort and cross sectional stepped wedge designs. Further research is needed on the effect of adjusting for the potential confounding of time on the study power.

## Background

Randomised Controlled Trials (RCTs) are the gold standard for evaluating the effectiveness of an intervention [1]. However sometimes individual randomisation is not convenient or in some situations even possible [2]. For example, some interventions can only be delivered at the group, community or organisational level whereas for other interventions there is the risk of contamination of individuals in the control arm with those in the intervention arm. Cluster randomised controlled trials (CRCTs) provide an approach to overcome these issues by randomising participants to either the intervention or control condition in groups rather than as individuals. The most common type of CRCT is referred to as a parallel design because clusters in each arm receive their respective intervention regime at the same time but independently of each other.

CRCTs have become very common in health related research and the statistical implications of conducting a study in this manner have been well documented [2–8]. The most important implication of randomising clusters instead of individuals is that observations from individuals within a cluster may be correlated (i.e. not independent). There are also circumstances where conducting a parallel CRCT is not possible. For some interventions the cost of simultaneous implementation to multiple clusters may be too high or logistically impractical [9, 10]. For other interventions, especially those already proven in individual level RCTs, some authors have argued that it is unethical to withhold the treatment from entire clusters [11, 12]. Withholding an intervention which has not yet proven to be effective is not considered to be unethical but this is often not the perception of individuals, organisations or units which may constitute the clusters for a CRCT, or even ethics committees. Consistent with this perception and the experience of the authors of this paper, recruitment of clusters such as hospitals is easier if the cluster is guaranteed to get the intervention at some stage [13].

**Fig. 1:**
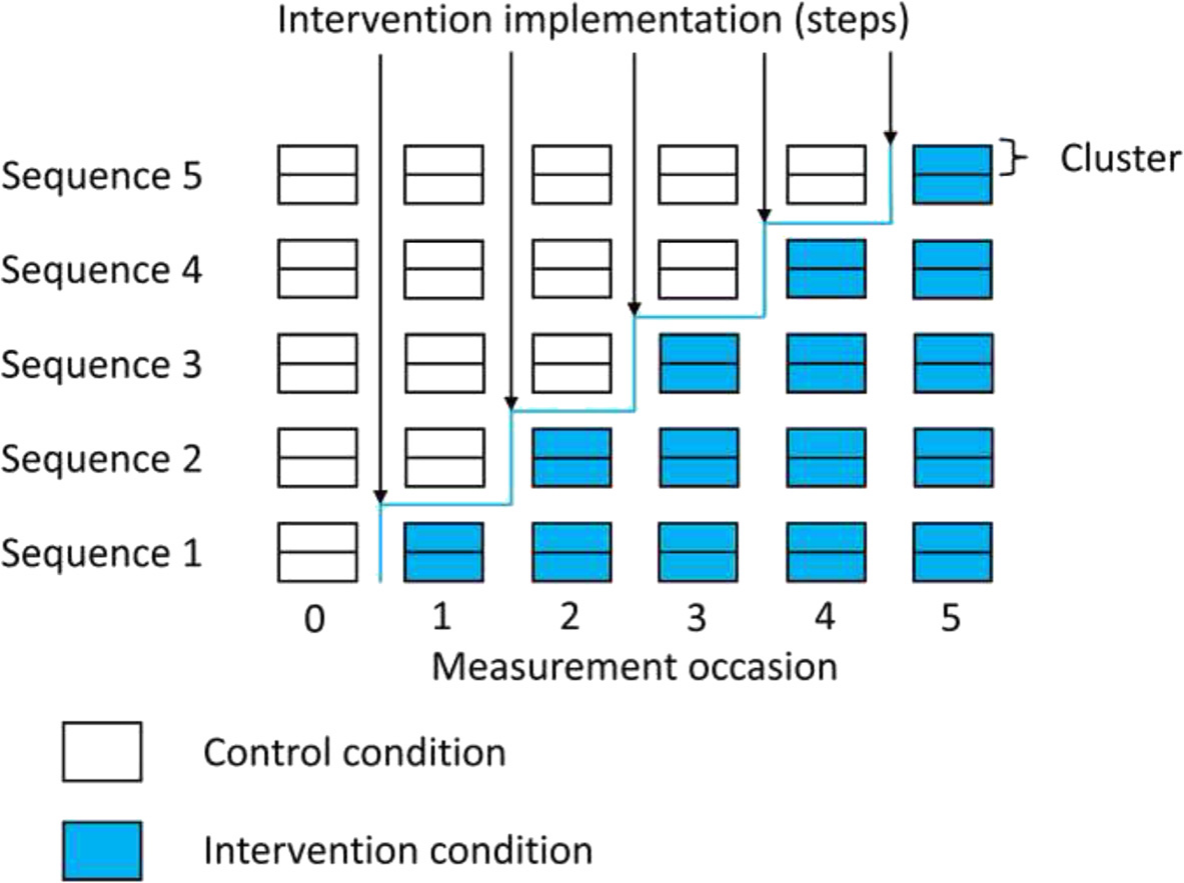
Trial Diagram of a possible stepped wedge design

The stepped wedge design (SWD) offers a potential solution to these logistical and ethical problems. In a SWD every cluster begins in the control condition and every cluster receives the intervention by the end of the study. This is achieved through the following process. Before the trial begins, the period of data collection is organised into two or more sequences of measurements. All of the sequences have the first measurement in the control condition and all have the last measurement in the intervention condition, but the time at which clusters within a sequence change from the control to the intervention condition is different for each sequence. Clusters are randomised to these sequences so that each sequence contains at least one cluster. Figure 1 contains a diagrammatic representation of a stepped wedge CRCT where the intervention is implemented in two clusters at each “step” which we define as a point in time where at least one cluster has changed from control to intervention condition. There is a measurement occasion on both sides of every step, and when there are enough steps the overall picture looks somewhat like a wedge, giving the design its name. Despite the fact that publications of trials using the SWD have increased rapidly since its first recognised use in Gambia in 1986 [9, 14] very little attention has been paid to the fact that there are two sub categories of the SWD which differ depending on how data on participants within clusters are obtained. If at each new measurement occasion outcomes are measured on a different randomly selected set of participants in each cluster then the study is a cross sectional SWD. If outcomes at each time point are obtained from the same participants within clusters the trial is a cohort SWD.

The SWD does have some drawbacks however. It may take longer to conduct than a parallel CRCT and almost always involves more measurements overall [15] which in the cohort SWD might result in a greater burden on the participants. Unlike the parallel CRCT, the SWD also requires that the chosen method of analysis adjusts for the potential confounding effect of time [11, 16–19]. So far there has been limited exploration of the methods of analysis in the context of the SWD, particularly with respect to the adjustment for time. Previous reviews have focussed on the rationale for employing the SWD, the areas of research which the SWD has been applied to, and have described the general characteristics of the design [9, 10, 20]. These reviews also reported that a variety of statistical methods have been used in the analysis of stepped wedge CRCTs but they have not addressed the appropriateness of methods used to calculate the sample size. Given the substantial increase in the use of this design in recent years the aim of this review is to describe the methodological literature on SWDs and how these methods have been applied in practice.

One option for analysing individual level data in either a parallel CRCT or a SWD is to use a generalised linear mixed model (GLMM). The term mixed arises because these models estimate both fixed effects, which are the deterministic part of the model forming the regression line, and random effects, which estimate the stochastic variation of individual clusters around the conditional mean of the clusters. Models of this type are interchangeably referred to as mixed, hierarchical, multilevel or random effects models [21] and for the purposes of this paper these will hereafter be referred to simply as GLMMs. Another popular approach to the analysis of individual level data from CRCTs and SWDs is to use the generalised estimating equation (GEE) framework [22]. Unlike GLMMs which model the variance and covariance arising from correlated data directly, the GEE method primarily aims to model the population averaged effects while accounting for the correlation indirectly. The drawback of this approach is that GEEs suffer from inflated type I error rates when there are too few clusters [23]. The fact that many SWDs involve only a few clusters seems to be overlooked in previous reviews as well as in current papers on methods of analysis and sample size calculation [20, 24]. As part of this review we discuss the implication of the small number of clusters on the analysis and sample size calculation. Specifically we aimed to investigate: 1) the statistical methods that are currently recommended for use for the analysis of stepped wedge CRCTs; 2) the methods currently recommended for sample size/power estimation; 3) which methods of analysis for SWDs have been used in practice, and 4) which methods of sample size/power estimation for SWDs have been used in practice.

## Methods

### Search strategy

In October 2013 the Medline, Embase, and Cochrane databases were searched for the terms step wedge, stepped wedge, staged introduction, phased implementation, staggered implementation, phased recruitment, stepwise recruitment and one way crossover. The resulting titles and abstracts were scanned for eligibility by a single reviewer. When more information was required the article was obtained. The search was updated in June 2015 and included the additional terms dynamic wait list, roll-out randomized, one directional cross-over and randomised multiple baseline design. Also added to this review are the papers from the series in August 2015 in the journal *Trials* which covered the SWD.

### Eligibility criteria

Papers were considered to be eligible for the review if they were written in the English language and were divisable into one of two categories. The first category, corresponding to aims 1) and 2), included papers which contained methodological details about the SWD itself that could be generalised to any such trial. Specifically papers had to include detailed coverage of either the pros and cons of choosing to use the SWD over other designs, variations of the SWD, settings in which the SWD was suitable, the methods of statistical analysis arising directly from the SWD or the methods for estimation of study power/sample size. The second category of papers, corresponding to aims 3) and 4), had to contain an example or protocol of a clustered trial using a SWD with at least two steps.

### Data extraction and analysis

From the methodological (first category) papers we recorded the names of the authors, the year of publication and the relevant issues arising from the SWD that were presented in detail. We also examined the reference lists of eligible papers for additional papers which the search strategy had missed.

From papers describing the conduct of a trial that used the SWD (second category) we extracted the names of the authors, the year of publication, the number of clusters (unit of randomisation) and the number of steps. Where possible we also obtained the method used for sample size estimation, the method used for analysis of the primary outcome and whether or not time was adjusted for in that analysis. In addition to this we categorised the study as either a cross-sectional or cohort SWD based on the definition above. We followed the PRISMA guidelines for reporting systematic reviews where relevant [25].

## Results

### Literature search yield

**Fig. 2:**
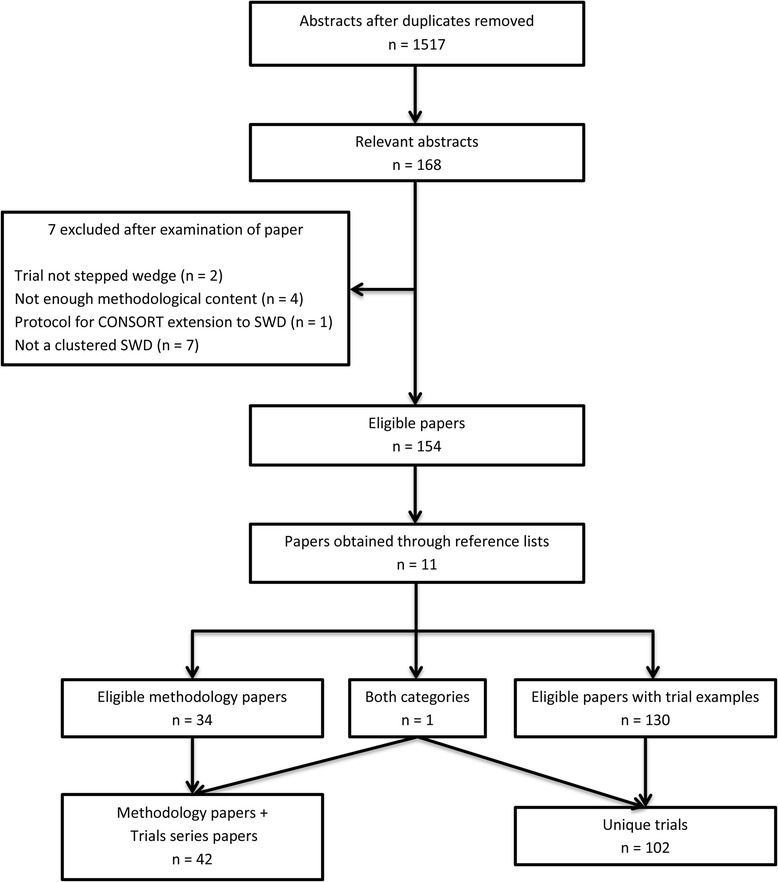
Results of the literature searchMethodology papers + Trials series papers *n* = 42

Figure 2 shows the literature search procedure described below. After removal of duplicates the search terms yielded a total of 1517 abstracts. 1349 of these were excluded because they were considered to be irrelevant. We excluded a further seven of the 168 remaining potentially eligible papers because they did not offer enough detail on the methodology related to the SWD and also did not provide an example of a stepped wedge trial. One of those excluded specified that the study was a stepped wedge trial in the abstract but no more detail was given about the study design, other than that it was a cross sectional intervention study. We identified an additional 11 papers from the reference lists of the 154 eligible papers. There were 42 papers eligible for the methodology category (as shown in Table 1), 130 papers which contained details of a trial implementing the SWD at the cluster level and one paper which was included in both categories [26]. From the 131 papers containing implementations of the SWD we identified 102 distinct trials (see Table 2).

**Table 1:** Characteristics of studies which considered methodological aspects (rationale, design or analysis) of Stepped Wedge Cluster Randomised Trials

First Author	Reasons for or against using the SWD	Variations of the SWD	Settings where the SWD can be applied	Method for estimating sample size	Method for statistical analysis
Bellan [93]	Yes	No	Yes	No	No
Beard/Lewis [94]	Yes	No (see Copas et al. instead)	Yes	No (see Baio et al. instead)	No (see Davey et al. instead)
Baio [38]	No	No	No	Yes (By simulation)	Yes (GLMM)
Brown [9]	Yes	No	Yes	No	No
Brown [19]	Yes	No	Yes	No	No
Brown [36]	Yes	No	Yes	Yes	No
Copas [43]	No	Yes	No	No	No
Davey [35]	No (see Bear/Lewis instead)	No	No	No	Yes (GLMM, Cox Proportional Hazards in cohort SWD setting)
de Hoop [41]	Yes	No	No	No	No
Fatemi [95]	Yes	Yes	Yes	No	No
Fok [27]	Yes	No	No	No	Yes (GLMM)
Gruber [28]	No	No	No	No	Yes (Causal Modelling)
Haines	Yes	Yes	Yes	No	No
Handley [11]	Yes	Yes	No	No	No
Hargreaves [96]	Yes	No (see Copas et al. instead)	Yes	No (see Baio et al. instead)	No (see Davey et al. instead)
Hargreaves [97]	No	Yes (Randomisation vs Non-randomisation	No	No	No
Hemming [17]	Yes	Yes	No	No	No
Hemming [42]	Yes	No	No	No	No
Hemming [24]	Yes	No	Yes	No	Yes (GLMM, GEE)
Hemming [37]	No	Yes	No	Yes (Extension of Hussey)	No
Hussey [29]	No	No	No	Yes (Wald Test)	Yes (GLMM, GEE with Jack-knife SE, Cluster Summaries)
Keriel-Gascou [98]	Yes	No	No	No	No
Kotz [99]	Yes	No	No	No	No
Kotz [90]	Yes	No	No	No	No
Kotz [44]	No	No	No	No	No
Medge [10]	Yes	No	Yes	No	No
Medge [100]	Yes	Yes	Yes	No	No
Medge [101]	Yes	No	No	No	No
Moulton [26]	No	No	No	Yes	Yes (GLMM, GEE, Cox Proportional Hazards)
Pater [102]	No	No	Yes	No	No
Prost [103]	Yes	No	Yes	No	No
Rhoda [15]	Yes	No	No	Yes (Based on Hussey)	No
Sanson-Fisher [104]	Yes	No	No	No	No
Schelvis [105]	Yes	No	Yes	No	No
Scott [30]	No	No	No	No	Yes (GEE with few clusters adjustment)
Van den Heuvel [31]	Yes	Yes	No	No	Yes (GLMM)
van der Tweel [106]	Yes	No	Yes	No	No
Viechtbauer [107]	Yes	No	No	No	No
Woertman [16]	No	No	No	Yes (Design Effect based on Hussey)	No
Wolkewitz [108]	No	No	Yes	No	No
Wyman [32]	Yes	No	No	No	Yes (GLMM)
Zhan [109]	Yes	No	Yes	No	No

**Table 2:** Summary of features from studies employing a stepped wedge design

First Author	Type of SWD	n clusters (n steps)	Statistical analysis method used for primary outcome (Distribution of outcome)	Adjusted for time	Sample size estimation	Publication phase
Aoun [110] Aoun [111] Aoun [112] Aoun [113] Austin [114] Grande [115]	Cohort	3 (3)	GLMM (Normal)	Yes	Yes: Cluster Design Effect Adjustment	Results
Lo [116]	Cross sectional	54 (3)	GLMM (Binomial)	Unknown	Unknown	Results
Bacchieri [47]	Cohort	42 (2)	GLM with RVE (Poisson)	Yes	Yes: No Clustering adjustment	Results
Badenbroek [76]	Cohort	40 (4)	Not Mentioned	Unknown	Yes: Cluster Design Effect Adjustment	Protocol
Bailet [117]	Cross sectional	174 (2)	GLMM (Normal)	Yes	No	Results
Bailey [77]	Cohort	4 (4)	Unknown	Unknown	Unknown	Results
Bailey [82]	Cross sectional	6 (6)	GEE Few clusters adjustment (Binomial)	Yes	Yes: Simulated	Results
Banga [78]	Cross sectional	4 (4)	Not Mentioned	Unknown	Yes: Woertman method	Protocol
Barton [59] Somerville [58]	Cohort	119 (2)	Mann–Whitney U/ANCOVA (Normal)	Yes	Yes: No Clustering adjustment	Results
Bashour [118]	Cross sectional	4 (4)	GLMM (Normal)	Yes	Yes: Hussey/Hughes method	Results
Bennett [119]	Cohort	15 (3)	GLMM (Normal)	No	Yes: Cluster Design Effect Adjustment	Protocol
Bernabe-Ortiz [120]	Cross sectional	6 (6)	GEE (Normal)	No	Yes: Hussey/Hughes method	Protocol
Bouwsma [51]	Cohort	9 (9)	Cox PH models with gamma random effects (Time to Event)	Yes	Yes: Hussey/Hughes method	Protocol
Brimblecombe [80]	Cross sectional	20 (4)	GLMM (Normal)	Yes	Yes: Simulated	Protocol
Brown [121]	Cross sectional	9 (5)	GEE (Binomial)	Unknown	Yes: Cluster Design Effect Adjustment	Protocol
Brownell [122]	Cohort	20 (5)	GLMM (Binomial)	Yes	No	Results
Chavane [123]	Cross sectional	10 (10)	GLMM (Binomial)	Unknown	Yes: Cluster Design Effect Adjustment	Protocol
Chinbuah [124] Chinbuah [125]	Cross sectional	114 (2)	GLMM (Poisson/Binomial)	No	Yes: Cluster Design Effect Adjustment	Results
Ciliberto [71] Patel [73]	Cohort	7 (6)	GLM (Binomial)/Unspecified Survival Analysis (Time to Event)	No	Yes: No Clustering adjustment	Results
Cowan [65]	Cross sectional	6 (3)	GLM fixed effect for cluster (Binomial)	Yes	Yes: Hussey/Hughes method	Protocol
Crain [126] Solberg [127]	Cohort	96 (5)	GLMM (Not specified)	Yes	No	Protocol
Craine [66]	Cross sectional	5 (5)	GLM fixed effect for cluster (Binomial)	Yes	Yes: Hussey/Hughes method	Results
Dainty [128] Morrison [129]	Cross sectional	32 (4)	GEE (Binomial)	Yes	Yes: Hussey/Hughes method	Results
De Allegri [130] Gnawali [131]	Cohort	33 (3)	GLMM (Binomial)	Yes	Yes: Cluster Design Effect Adjustment	Results
Dilworth [61] Turner [132]	Cross sectional	5 (5)	GEE/Discourse Mapping (Normal)	No	Yes: Cluster Design Effect Adjustment	Protocol
Doherty [55]	Cross sectional	55 (2)	*χ* ^2^ test for trend (Multinomial)/Kruskal-Wallis (Nonparametric)	No	Yes: Details not clear	Results
Dreischulte [133]	Cohort	40 (10)	GLMM (Binomial)	Yes	Yes: Details not clear	Protocol
Dryden-Peterson [134]	Cross sectional	20 (10)	GEE (Binomial)	Yes	Yes: Details not clear	Results
Due [53]	Cohort	189 (2)	*T*-test (Normal)	No	Yes: Details not clear	Results
Duijster [135] Monse [136]	Cohort	13 (2)	GLMM (Normal)	Yes	No	Results
Durovni [49] Golub [50] Moulton [26]	Cross sectional	29 (14)	GLM with Scale Parameter (Poisson)/Cox PH with either Bootstrap SE, RVE or gamma random effect (Time to event)	Yes	Yes: Moulton	Results
Durovni [137] Trajman [138]	Cross sectional	14 (7)	GLMM (Poisson and Binomial)	Yes	Yes: Moulton	Results
Emond [139]	Cross sectional	9 (3)	GLMM (Normal and Binomial	Yes	Yes: Hussey/Hughes method	Protocol
Enns [140]	Cross sectional	4 (4)	GEE (Poisson)	Yes	No	Results
Etchells [141]	Cross sectional	2 (2)	GLMM (Normal and Binomial	No	No	Results
Fernald [142]	Cohort	506 (2)	GLM adjusting for sampling design and clustering (Normal and Binomial)	No	Yes: Cluster Design Effect Adjustment	Results
Franklin [143]	Cross sectional	15 (5)	GLMM (Binomial)	Yes	Yes: Details not clear	Results
Fuller [81]	Cross sectional	60 (5)	GLMM (Binomial)	Yes	Yes: Simulated	Results
Gambia Hepatitis Study Group [14]	Cross sectional	17 (16)	Not Mentioned	Unknown	Yes: No Clustering adjustment	Protocol
Gerritsen [144] Leontjevas [145] Leontjevas [146] Leontjevas [147]	Cohort	5 (5)	GLMM (Normal and Binomial)	Yes	Yes: Hussey/Hughes method	Results
Golden [34]	Cross sectional	23 (4)	GLMM/GEE (Binomial/Poisson)	Yes	Yes: Hussey/Hughes method	Results
Groshaus [67]	Cohort	6 (6)	GLM fixed effect for cluster (Normal and Binomial)	No	No	Results
Gruber [148]	Cohort	24 (6)	GEE with RVE (Binomial)	Yes	Yes: Hussey/Hughes method	Results
Grunewaldt [54]	Cohort	20 (2)	*χ* ^2^ tests (Binomial)/t-tests (Normal)	No	No	Results
Gucciardi [149]	Cross sectional	4 (3)	GLMM/GEE (Binomial)	Yes	Yes: Cluster Design Effect Adjustment	Protocol
Haines [150]	Cross sectional	6 (6)	GLMM (Normal)	Yes	Yes: Hussey/Hughes method	Protocol
Haugen [74]	Cross sectional	5 (5)	GLM (Binomial)	Yes	Yes: Details not clear	Results
Haukka [151]	Cross sectional	9 (4)	Unspecified Survival Analysis (Time to event)/GEE (Normal)	No	No	Protocol
Hayden [152]	Cohort	4 (4)	GLMM (Binomial)	Yes	Yes: Details not clear	Results
Hill [153] Hill	Cross sectional	8 (4)	GLM RVE (Negative Binomial and Binomial)	Yes	Yes: Cluster Design Effect Adjustment	Results
Horner [154]	Cohort	65 (3)	GLMM (Binomial)	Yes	No	Results
Howlin [155]	Cohort	18 (2)	GLMM (Ordinal)	No	Yes: Cluster Design Effect Adjustment	Results
Hunter [156]	Cross sectional	8 (2)	GLMM (Binomial)	No	Yes: Cluster Design Effect Adjustment	Protocol
Husaini [157]	Cohort	45 (2)	GLMM (Binomial)	No	No	Results
Kelly [68]	Cross sectional	4 (4)	GLM fixed effect for cluster or Bootstrap SE (Normal)	Yes	Yes: Cluster Design Effect Adjustment	Protocol
Keriel-Gascou [98]	Cross sectional	8 (4)	GLMM (Binomial)	Yes	Yes: Hussey/Hughes method	Protocol
Killam [158]	Cross sectional	8 (8)	GEE (Binomial)	Yes	No	Results
Kitson [159] Schultz [160]	Cross sectional	25 (4)	GLMM (Normal)	Yes	Yes: Hussey/Hughes method	Results
Kjeken [161]	Cohort	6 (6)	GLMM (Normal)	Yes	Yes: Cluster Design Effect Adjustment	Protocol
Larsen [46]	Cross sectional	46 (18)	GLM with RVE (Binomial)	Yes	Yes: Details not clear	Results
Li [162]	Cross sectional	104 (4)	GEE (Binomial)	Yes	Yes: Cluster Design Effect Adjustment	Protocol
Liddy [163]	Cohort	83 (3)	GLMM (Normal)	Yes	Yes: Cluster Design Effect Adjustment	Protocol
Lilly [87]	Cross sectional	7 (7)	GLMM (Binomial and Normal)	Yes	Yes: No Clustering adjustment	Results
Maheu-Giroux [164]	Cross sectional	6 (3)	GLMM (Binomial)	Yes	No	Results
Marrin [165]	Cross sectional	6 (3)	GLMM (Normal)	No	Yes: Cluster Design Effect Adjustment	Protocol
Marshall [166]	Cross sectional	32 (10)	GEE (Binomial)	Yes	Yes: Cluster Design Effect Adjustment	Protocol
Mhurchu [167] Mhurchu [168]	Cohort	14 (4)	GLMM (Binomial and Normal)	Yes	Yes: Hussey/Hughes method	Results
Mosha [72]	Cross sectional	10 (10)	GLM (Binomial)	No	No	Results
Mouchoux [62]	Cross sectional	3 (3)	GLM fixed effect for cluster (Binomial)	Yes	Yes: Hussey/Hughes method	Protocol
Muntinga [169]	Cohort	35 (4)	GLMM (Normal)	Yes	Yes: Cluster Design Effect Adjustment	Protocol
Ononge [75]	Cross sectional	6 (Unclear)	GLM (Normal and Binomial)	No	No	Results
Palmay [170]	Cross sectional	6 (6)	GLMM (Negative binomial)	Yes	No	Results
Palmer [83]	Cohort	11 (3)	GLMM (Normal)	Yes	Yes: Simulated	Protocol
Pearse [52]	Cross sectional	90 (15)	GLMM (Binomial)/Cox PH (Time to Event)	Yes	Yes: Hussey/Hughes method	Protocol
Pickering [171]	Cohort	4 (4)	GLMM (Normal)	Yes	Yes: Details not clear	Results
Poldervaart [172]	Cross sectional	10 (10)	GLMM (Binomial)	No	Yes: Cluster Design Effect Adjustment	Protocol
Priestley [88]	Cross sectional	16 (7)	GLMM (Binomial)	Unknown	Yes: No Clustering adjustment	Results
Proeschold-Bell [79]	Cohort	3 (3)	Unknown	Unknown	No	Protocol
Rasmussen [173]	Cohort	21 (4)	GLMM (Normal)	No	Yes: Woertman method	Results
Reuther [174]	Cohort	12 (5)	GLMM (Binomial)	Yes	Yes: Hussey/Hughes method	Protocol
Roy [175]	Cross sectional	27 (5)	GLMM (Binomial)	No	Yes: Cluster Design Effect Adjustment	Results
Schnelle [60]	Cohort	2 (2)	Repeated Measures ANOVA (Normal)	Yes	Unknown	Results
Skrovseth [57]	Cohort	2 (2)	GLM (Normal)/Wilcoxon Rank sum (Non parametric)	No	Yes: Details not clear	Results
Solomon [176] Solomon [177]	Cross sectional	128 (4)	GLMM/GEE (Normal/Binomial)	Yes	Yes: Hussey/Hughes method	Results
Stern [84]	Cohort	12 (11)	GLMM (Normal)	Yes	Yes: Simulated	Results
Strijbos [89]	Cross sectional	8 (4)	GLMM (Binomial)	No	Yes: No Clustering adjustment	Protocol
Suman [178]	Cohort	4 (4)	GLMM (Binomial)	No	Yes: Cluster Design Effect Adjustment	Protocol
Tielsch [85]	Cohort	12 (12)	GLMM (Normal and Binomial)	Yes	Yes: Simulated	Protocol
Tiono [179]	Cohort (Cross sectional subgroup)	40 (8)	GLMM (Binomial)	Yes	Yes: Cluster Design Effect Adjustment	Protocol
Tirlea [180] Tirlea [181]	Cohort	12 (3)	GLMM (Normal)	Yes	Yes: Cluster Design Effect Adjustment	Results
Toftegaard [182]	Cohort (GP level) Cross Sectional (patient level)	8 (8)	GLMM (Binomial)	No	Yes: Woertman method	Protocol
Viera [56]	Cohort	3 (2)	McNemar’s test (Binomial)	No	Retrospectively: No clustering adjustment	Results
Ward [183]	Cross sectional	24 (3)	GLMM/GEE (Binomial)	No	Yes: Cluster Design Effect Adjustment	Protocol
Weiner [69]	Cross sectional	36 (36)	GLM fixed effect for cluster (Binomial)	No	No	Results
Williams [184]	Cohort	12 (3)	GLMM (Normal)	Yes	Yes: Hussey/Hughes method	Protocol
Williamson [70]	Cohort	6 (3)	GLM fixed effect for cluster (Binomial and Normal)	Yes	Yes: Hussey/Hughes method	Protocol
Wilrycx [185]	Cohort	2 (2)	GLMM (Normal)	Unknown	No	Results
Zwijsen [186] Zwijsen [187] Zwijsen [188] Zwijsen [189]	Cohort	17 (5)	GLMM (Binomial)	No	Yes: Hussey/Hughes method	Results
van Daalen [86]	Cross sectional	9 (4)	GLMM/GEE (Normal/Binomial)	No	Yes: Simulated	Protocol
van de Steeg [190] van de Steeg [191]	Cross sectional	18 (10)	GLMM (Binomial)	Yes	Yes: Cluster Design Effect Adjustment	Results
van den Broek [63] van den Broek [64]	Cross sectional	190 (3)	GLM fixed effect for cluster (Binomial)	No	Yes: Simulated	Results
van Holland [192]	Cohort	5 (5)	GLMM (Normal and Binomial)	Yes	Yes: Hussey/Hughes method	Protocol

### Methodological papers

A detailed examination of the statistical analysis approach for SWD was provided by 10 of the 42 papers which covered methodology [24, 26–32]. The paper by Hussey and Hughes [29] is the most widely cited of these and specifies a GLMM with a random intercept for clusters as the recommended method of analysis; however this applies only to the cross-sectional SWD. Hussey and Hughes also suggest that a GEE or a linear mixed model of the cluster summaries can be used and it is a noteworthy feature of this paper that the analysis methods were examined in both the equal cluster size and unequal cluster size scenarios. Both the GEE and GLMM models that Hussey and Hughes examined used the jack-knife variance estimate [33] instead of the robust variance estimator (RVE) because of the limited number of clusters in their motivating example, which was a trial planned with 24 county health districts in Washington state [34]. Scott et al. [30] suggest that a GEE is most appealing because of the marginal interpretation of model parameters and the lack of assumptions required about the latent variable distributions which are specified as random effects in GLMM. The defining feature of the Scott et al. paper is the comparison of four methods of small sample (small because of few clusters) corrections to the GEE modelling approach. Moulton et al. [26] outline an approach to the analysis of a SWD using a Cox proportional hazards model adjusting for clustering by either bootstrapping or using the RVE. Other authors also suggested the use of a GLMM or a GEE [24, 26, 27, 31, 32, 35] but some had different views on how the fixed or random effects should be specified. For example both Wyman et al. [32] and Van den Heuvel et al. [31] advocate the use of random effects for time in addition to the random intercept whereas Fok et al. [27] outline a random intercept model with an interaction term between time and intervention as a fixed effect. Gruber et al. take a causal modelling approach to the analysis and outline how to estimate the complier average causal effect from a stepped wedge trial [28]. They also note that the SWD has the advantage over parallel CRCTs in the testing of identification assumptions required to use the instrumental variables estimator. Finally Davey et al. use 3 examples (2 GLMMs and a Cox PH model) to explore potential analyses of cohort SWDs [35]. They also discuss the possible drawbacks that many of the simpler models possess; the secular trend and intervention effect is the same for all clusters. To that end they discuss the potential for additional random effects, particularly a random intervention by cluster term. However they do not discuss the potential analysis problems associated with smaller SWDs, such as the lack of statistical power required to fit the more complex models being suggested.

There were seven papers which offered a method of calculating power or sample size [15, 16, 26, 29, 36–38]. Hussey and Hughes suggest a method for calculating the study power of a cross sectional stepped wedge design based on a Wald test of the intervention effect from a weighted least squares analysis [29]. Hemming et al. extend the Hussey and Hughes method to several variations of the cross sectional SWD such as those which do not measure the outcome from every cluster at every step [37]. This generic method also encompasses the parallel CRCT and variations of it so that comparing the power of different cross sectional designs is more straightforward. Hemming and Girling also produced the user written program *steppedwedge* in the software package Stata [39] which can calculate the power for a variety of SWDs with continuous, binary or rate outcomes [40]. Woertman et al. proposed a formula for the design effect of a SWD based on the Hussey and Hughes method which when multiplied by the estimated sample size required for an individually randomised trial would yield the required number of subjects in the SWD [16]. Unfortunately this method applied the cross sectional SWD model to a cohort SWD trial and is potentially misleading because the correlation within subjects over time is therefore assumed to be zero, which is unlikely [41, 42]. Rhoda et al. also build on the work by Hussey and Hughes to derive a formula for the variance of the intervention effect estimator for both the SWD and a parallel CRCT with the same number of measurement times [15]. They take the ratio of these variances to compare the power of the two designs and present the conditions in terms of the number of steps and the values of the intra-cluster correlation (ICC) for which the SWD is more powerful than the parallel CRCT and vice versa. Moulton et al. outline a method in which the design effect is calculated based on the log-rank test statistic using information about the coefficient of variation (CV) of the outcome, which they obtained from pilot data [26]. Brown et al. present a power calculation based on a GLMM with a Poisson distributed outcome that has a random intercept and random slope for time [36]. Finally Baio et al. present a simulation based procedure for sample size estimation [38]. These authors raise some very important issues about sample size estimation in stepped wedge trials that have not been discussed anywhere else. The first issue is that existing methods do not account for secular trends and the failure to do so when such a trend exists overestimates study power. The second important point is that additional random effects, such as a random intervention effect, decrease study power considerably.

Twenty eight of the 42 papers included in the methodology category did not include any detail on the methods for analysis or sample size estimation. The most informative of these is the paper by Copas et al. [43]. These authors discuss the key features of the SWD in detail and defined 3 types of SWD based on the recruitment of patients within clusters. These are the cross-sectional, closed cohort and open cohort SWDs. Other papers discuss how the SWD potentially increases statistical power [16, 36, 41, 42, 44] while some discuss the need to adjust for the potential confounding effect of time [11, 16–19]. Further papers discussed broader aspects of the SWD such as the motivations for its use [19, 42, 45], the advantages and disadvantages of its use, or the possible settings for which the SWD is suited (see Table 1).

### Examples of stepped wedge cluster RCTs

Consistent with three previous reviews of the SWD [9, 10, 20] the number of steps in the identified studies ranged between 2 and 36 with a median of 4. The number of clusters randomised varied even more, ranging from between 2 and 506. In contrast to the latest review [20] which found the median number of clusters per trial was 17 we found that the median was 12 and indeed most trials were modest in terms of the number of clusters with 64 % (*n* = 65) of the studies sampling fewer than 20 clusters and almost half (*n* = 46, 45 %) sampling fewer than 10. There were slightly more (*n* = 56, 55 %) studies which sampled participants in a cross sectional manner whereas the remainder were designed such that participants were repeatedly sampled as in a cohort SWD. Publications of trials using the SWD have increased rapidly in the past few years; more than three quarters (*n* = 101/131) of the papers in Table 2 were published since the beginning of 2012.

### Method of analysis

The method of statistical analysis used varied between studies. Whilst it is not clear if this is due to a lack of agreement on how to analyse the data from a SWD or because of the variety of applications of the SWD or some combination of the two, the majority of studies chose to adjust for the longitudinal nature of the SWD with either GLMMs (*n* = 60, 59 %) or GEEs (*n* = 17, 17 %). The remainder used a variety of methods including generalised linear models (GLM) with robust variance estimators [46–48], Cox proportional hazards modelling [26, 49–52], paired t-tests [53, 54], *χ*^2^ tests [54, 55], McNemar’s test [56], Wilcoxon rank sum test/Mann-Whitney *U* test [57, 58], Analysis of covariance (ANCOVA) [58, 59], Analysis of variance (ANOVA) [60], Discourse mapping [61], GLM’s with cluster as a fixed effect [62–70] and GLM’s without any reported effort to adjust for clustering [71–75]. For some studies the method of analysis was unclear [14, 76–79]. The potential confounding effect of time was explored in 61 of the 102 (60 %) studies and either adjusted for in the primary analysis or found not to be correlated with the outcome. For the remaining studies time was not mentioned in the context of confounding and it is unknown whether or not this factor was adjusted for.

### Sample size

Of the 79 (77 %) studies which did estimate sample size prospectively, 27 (26 %) of these did so by first determining the design effect based on the ICC as if the study were a parallel CRCT. The method outlined by Hussey and Hughes [29] was used in 22 (22 %) studies and the design effect method proposed by Woertman [16] was used in 3 studies. However 11 of these studies were cohort SWDs and these methods only apply to the cross sectional SWD. There were eight studies which simulated the power based on the method of analysis chosen [63, 64, 80–86] whilst one study used the coefficient of variation from pilot data to estimate the required sample size [26]. Seven studies calculated sample size without consideration for the effect of clustering [14, 47, 58, 59, 71, 73, 87–89]. There was one study which calculated the sample size retrospectively [56] and 22 (22 %) which either did not include details of, or did not estimate the sample size.

## Discussion

This review confirmed that use of the SWD has increased substantially in recent years [20]. Reasons for the increased use have been speculated on in the past [10] but one appealing advantage which is often cited, is the ability of the SWD to maintain the same power with fewer clusters than the parallel CRCT [16, 36, 41, 78, 90]. However this is only true when the comparison is between the parallel CRCT with a single measurement period and the SWD with its multiple measurements. When both the parallel CRCT and the SWD have the same number of cross-sectional measurements, the latter is more powerful only when there are a sufficient number of steps [15]. It is unknown if this is the case for the cohort SWD and given the high proportion of cohort SWD studies (45 %) this is an area which needs more research. We would also like to point out that the ethical justification for the SWD may differ between the cohort and cross sectional SWD. All clusters will eventually receive the intervention in both designs but only half the participants will receive the intervention in a balanced cross-sectional SWD, since cluster members will be in the control phase until after the intervention has been implemented in their cluster. Thus even in a SWD the intervention can be withheld from participants. Only the cohort SWD will guarantee that all participants eventually receive the intervention.

Broadly speaking the recommended method of statistical analysis is a choice of either a GLMM or a GEE, both of which have a long history of use in parallel cluster RCTs. To date there has been little investigation into the minimum number of clusters required for a SWD. This is particularly important since this review demonstrated that the stepped wedge design was often used with a small number of clusters. Nearly half of stepped wedge cluster RCTs had fewer than 10 clusters which is a perilously low number for both unbiased estimation and statistical power for each of the recommended methods of analysis. For example the variance of the regression estimates from a GEE using only the robust variance estimator will generally be underestimated when the number of clusters is fewer than 50 [91]. It is possible to reduce the number of clusters needed if the correlation structure is correctly specified, but in general this structure is not known. Even the small sample methods such as the jack-knife method require 20 or more clusters to estimate the variance of the intervention effect with enough precision [91] and Scott et al. found only one of the methods of small sample adjustment which maintained coverage with as few as 10 clusters [30]. GLMM models also require a sufficient number of clusters in order to estimate the random effects. Snijders and Bosker [92] suggest that these models not be used when the number of clusters is fewer than 10 and results from the simulations of Baio et al. [38] suggest that under ideal circumstances a GLMM with only a random intercept might require as few as 8 clusters if the outcome is a count with a Poisson distribution. However as the number of cluster level random effects included in the model increases so too does the number of clusters required for adequate estimation. Although we agree with the rationale for the strong recommendation by Davey et al [35] that a “random intervention by cluster term” be added to the analysis, we must point out that many SWDs do not have a sufficient number of clusters to estimate this additional random effect accurately. For other methods of analysis, such as the cluster summaries method proposed by Hussey and Hughes [29], it is unknown how many clusters are required as a minimum. We suggest that researchers be aware that there will be a lower limit because the gain in power associated with more measurement periods diminishes as the number of measurement times increases [29], whereas reducing the number of clusters results in a relatively large loss of power for smaller trials. More simply put, between cluster information cannot be traded for within cluster information ad infinitum. While the lower limit to the number of clusters for a SWD is unknown, it will depend on several factors including whether the model is linear or non-linear and how balanced the clusters are in terms of size.

Time is another factor that must be considered in the analysis of a SWD because the study design itself induces an association between time and the intervention. If there is also an association between time and outcome, either directly or due to other predictors of the outcome changing over time then this meets the definition of a confounder. Many authors have correctly pointed out that the method of analysis needs to adjust for time [11, 16–19] but the impact of doing so on the statistical power of the analysis has only been investigated by Baio et al. [38]. These authors have found that adjusting for a time effect results in a loss of statistical power. This needs to be accounted for in future sample size estimates for SWDs but at present the best method of estimating the required sample size for a SWD, particularly the cohort design is to simulate the power based on the method of analysis proposed while also including time (as a fixed or random effect) in the chosen model.

## Conclusions

Statistical methodology for SWDs continues to lag behind what is implemented in practice. Many SWDs might also be underpowered or even biased because they contain too few clusters for the chosen method of analysis. Further research is needed on the minimum number of clusters required to conduct both types of SWD, as well as on the most appropriate method of analysis for stepped wedge CRCTs when there are few clusters. Another methodological deficiency is the lack of research for the cohort SWD, and the lack of a clear distinction between it and the cross-sectional SWD to the point where trialists are confusing the methods of sample size calculation for cross-sectional SWDs and cohort SWDs. Researchers need to be aware that the cohort and cross sectional SWD require different approaches to sample size estimation and that the SWD should not be employed solely on the basis of a ‘simple’ power estimate which takes no account of the complex design. There is also need for further research on the effect of adjusting for time on the study power and therefore sample size estimates, which will impact the studies with small numbers of clusters the greatest. Finally we point out that it is yet to be proven if the SWD is more powerful than an equivalent parallel CRCT when all of the complexities of the design, such as time adjustment, are accounted for in the statistical analysis.

## Abbreviations

ANCOVA, ANalysis of COVAriance; ANOVA, ANalysis Of VAriance; CRCT, cluster randomised controlled trial; GEE, generalised estimating equation; GLM, generalised linear model; GLMM, generalised linear mixed model; ICC, intra-cluster correlation; RCT, randomised controlled trial; RVE, robust variance estimator; SWD, stepped wedge design
